# Long-term outcome of natalizumab-associated progressive multifocal leukoencephalopathy in Austria: a nationwide retrospective study

**DOI:** 10.1007/s00415-023-11924-7

**Published:** 2023-09-20

**Authors:** Tobias Moser, Georg Zimmermann, Anna Baumgartner, Thomas Berger, Gabriel Bsteh, Franziska Di Pauli, Christian Enzinger, Elisabeth Fertl, Thomas Heller, Stefan Koppi, Paulus S. Rommer, Georg Safoschnik, Thomas Seifert-Held, Robert Stepansky, Johann Sellner

**Affiliations:** 1 Department of Neurology, Christian Doppler University Hospital, Paracelsus Medical University, Salzburg, Austria; 2 Team Biostatistics and Big Medical Data, IDA Lab Salzburg, Paracelsus Medical University, Salzburg, Austria; 3 Department of Research and Innovation, Paracelsus Medical University, Salzburg, Austria; 4 Department of Neurology, Comprehensive Center for Clinical Neurosciences and Mental Health, Medical University of Vienna, Vienna, Austria; 5 Department of Neurology, Medical University of Innsbruck, Innsbruck, Austria; 6 Department of Neurology, Medical University of Graz, Graz, Austria; 7 Department of Neurology, Klinik Landstrasse, Vienna, Austria; 8 Rehabilitation Clinic Montafon, Schruns, Austria; 9 Private Practice, Baden, Lower Austria Austria; 10 Department of Neurology, Landeskrankenhaus Murtal, Knittelfeld, Austria; 11 Department of Neurology, St John’s Hospital, Vienna, Austria; 12 Department of Neurology, Landesklinikum Mistelbach-Gänserndorf, Liechtensteinstrasse 67, 2130 Mistelbach, Austria

**Keywords:** Multiple sclerosis, Human polyomavirus 2, Progressive multifocal leukoencephalopathy, Natalizumab, Long-term outcome, Immunotherapy

## Abstract

**Background/objective:**

The use of natalizumab (NAT) in multiple sclerosis (MS) may be complicated by progressive multifocal leukoencephalopathy (PML), a rare and life-threatening opportunistic brain infection. We aimed to analyze the course of MS after PML recovery together with the long-term outcome of NAT-associated PML (NAT-PML) in Austria.

**Methods:**

Retrospective study based on identification of cases in the nationwide Austrian MS treatment registry (AMSTR) and MS centers with review of patient records. The expanded disability status scale (EDSS) was used to measure neurological disability and outcome.

**Results:**

As of December 2022, we identified 15 NAT-PML cases in Austria; only 20% occurred after 2016, when increased vigilance commenced. Two patients did not survive acute PML, and an additional patient died five years later, yielding a mortality rate of 20%. Seizures occurred exclusively in patients with pronounced EDSS increase. Gadolinium (Gd)-enhancement on brain magnetic resonance imaging (MRI) on PML suspicion was associated with minor changes of post-PML neurological disability. Long-term follow-up of up to 132 months (median 76 months) was available in 11/15. The overall median EDSS increased from 3.5 at pre-PML to 6.5 at the last assessment. Regarding inflammatory MS-related disease activity during the observation period, one single individual experienced an MS relapse and another patient had two Gd-enhancing brain lesions. Three patients converted to progressive MS within three years from PML and the EDSS further increased in 6/11.

**Conclusions:**

The number of NAT-PML cases is decreasing over time. While many patients accumulated severe persistent neurological deficits compared to pre-PML, inflammatory MS-related disease activity after PML recovery was rare.

**Supplementary Information:**

The online version contains supplementary material available at 10.1007/s00415-023-11924-7.

## Introduction

Natalizumab (NAT, Biogen Idec Inc, Cambridge, Massachusetts, USA) is a monoclonal antibody that was approved in 2004 for the treatment of active relapsing–remitting multiple sclerosis (MS) [1]. NAT blocks the alpha4-beta1 integrin receptor on lymphocytes and thereby prevents migration of immune cells into the brain [1, 2]. Its use carries a uniquely high risk for the development of progressive multifocal leukoencephalopathy (PML), an opportunistic infection caused by *human polyomavirus 2*, also known as JC polyomavirus (JCV) [3, 4]. NAT-associated PML (NAT-PML) is a potentially fatal condition that causes substantial morbidity among survivors [5, 6]. Primary infection with JCV is asymptomatic, and the seropositivity in the general population is as high as 50–80% and increases with age [7, 8]. Despite the widespread JCV exposure, the prevalence is < 10 per 1000 NAT-treated patients [9]. Factors that increase the risk of NAT-PML include prior treatment with immunosuppressants, long-term NAT use, and high JCV antibody index [10, 11]. Along with the presence of PML compatible clinical signs, that vary depending on the brain area affected, the diagnostic workup consists of cerebral magnetic resonance imaging (MRI) findings suggestive of PML and importantly, the demonstration of JCV DNA in the cerebrospinal fluid (CSF) [12, 13]. The management of NAT-PML relies on NAT removal to restore immune surveillance in the central nervous system (CNS) and is, if implemented early, associated with a better outcome [14, 15]. While several studies have assessed the acute phase of the disease, few investigations exist on the clinical course of patients after surviving PML. Also, reports exploring the inflammatory and neurodegenerative disease course in the aftermath of NAT-PML are lacking and the post-PML immunomodulatory treatment strategy remains a matter of debate. The ideal timing for the initiation of immunomodulation following a severe brain infection is challenging as a potential interference with the immune response to JCV must be weighed against the risk of MS exacerbation. The main objective of this study was to analyze the clinical course of MS after NAT-PML recovery and to characterize the long-term outcome and immunomodulatory treatment approaches in a nationwide cohort.

## Methods

### Data collection

In this retrospective study, we identified NAT-PML cases from a query within the database of the Austrian MS Treatment Registry (AMSTR). The AMSTR was established in 2006 after occurrence of the first NAT-PML cases worldwide. It represents a quality control tool to assess real-world safety and effectiveness of disease modifying therapies (DMTs) in MS. Prescription and reimbursement of DMTs in Austria is exclusively reserved for certified MS centers, which are obliged to participate in the registry. Moreover, Austrian MS centers were directly contacted for identification of further NAT-PML patients. After ascertaining the NAT-PML cohort, the treating physicians were contacted to provide demographics, MS and PML disease characteristics, risk factors for NAT-PML including previous immunosuppressive therapies laboratory findings, neuroimaging data and therapeutic interventions. Moreover, we studied the clinical course, MRI data and the immunomodulatory treatment regime throughout December 2022. Data were collected and analyzed on the basis of an excel worksheet. The expanded disability status scale (EDSS) was used to determine the neurological short- and long-term outcome and was assessed before PML (“pre-PML” defined as the last EDSS available before the PML diagnosis, assessed within one year from PML onset), after PML (“post-PML” refers to the lowest EDSS reached after recovery from PML, but no longer than one year from PML diagnosis) and at the end of the observation period (“long-term” refers to the last available EDSS score in 2022). EDSS changes from pre- to post-PML determined short-term outcome and EDSS changes from pre-PML to long-term characterized the long-term outcome. According to the EDSS increase, we stratified the functional short- and long-term outcome into mild (≤ 1.5) and severe (> 1.5) disability accumulation in order to evaluate clinical deterioration directly associated with PML and the clinical progression observed during long-term follow-up after PML recovery. For patients that were lost to final follow-up, the most recent EDSS available after PML recovery was used to determine outcome. In addition to the EDSS, we also assessed the modified Rankin scale (mRS) at the end of the observation period. The mRS is a measure of disability in the daily activities developed for stroke patients which ranges from 0 (asymptomatic) to 6 (death). Inflammatory MS-related disease activity after PML recovery included clinical relapses or new or enlarging T2 lesions or gadolinium (Gd)-enhancing T1 lesions on MRI. Transition to secondary progressive MS (SPMS) was evaluated by the respective treating physicians. We used the American Academy of Neurology (AAN) criteria to categorize “definite,” “probable,” and “possible PML” [12]. We stratified initial MRI findings into unilobar (if only one lobe was involved), infratentorial (only infratentorial involvement) and widespread (both hemispheres affected or one hemisphere plus an infratentorial involvement) affection. Moreover, we examined the first cerebral MRI scan performed at the time of PML suspicion for Gd enhancement, being suggestive of an inflammatory PML. The diagnosis of immune reconstitution inflammatory syndrome (IRIS) was based on clinical worsening as a correlate of immune reconstitution associated with radiological features such as new or expanding lesions with oedema and Gd enhancement on follow-up MRIs after the diagnosis of NAT-PML [16]. The presence of anti-JCV antibodies in serum or plasma and the corresponding JCV antibody index statuses were evaluated at pre-PML and, if available, after recovery from PML. A JCV antibody index of 0.4 represents the cut-off for JCV antibody positivity, while values below this level require an additional confirmation test [17]. One patients’ PML-IRIS course had been published as a case report before [18].

### Statistics

Data are shown as mean with standard deviation (SD) or median with interquartile range [IQR] depending upon normal distribution unless otherwise specified. Descriptive statistics were used to explore associations between long-term EDSS change and the following parameters: demographics, diagnostic delay, and risk factors (number of NAT infusions, JCV antibody index), presenting symptoms, MRI findings, occurrence of IRIS or seizures during PML, and usage of plasma exchange. The figures were created with BioRender.com.

### Ethics approval

The study protocol was evaluated by the local Ethics Committee (Ethikkommission für das Bundesland Salzburg; 415-EP/73/534-2015). We received a waiver for patient consent due to the retrospective, noninterventional design using anonymized data. This study was conducted according to the ethical principles of the Declaration of Helsinki and did not interfere with the care received by patients.

## Results

### Demographics and risk stratification

**Fig. 1:**
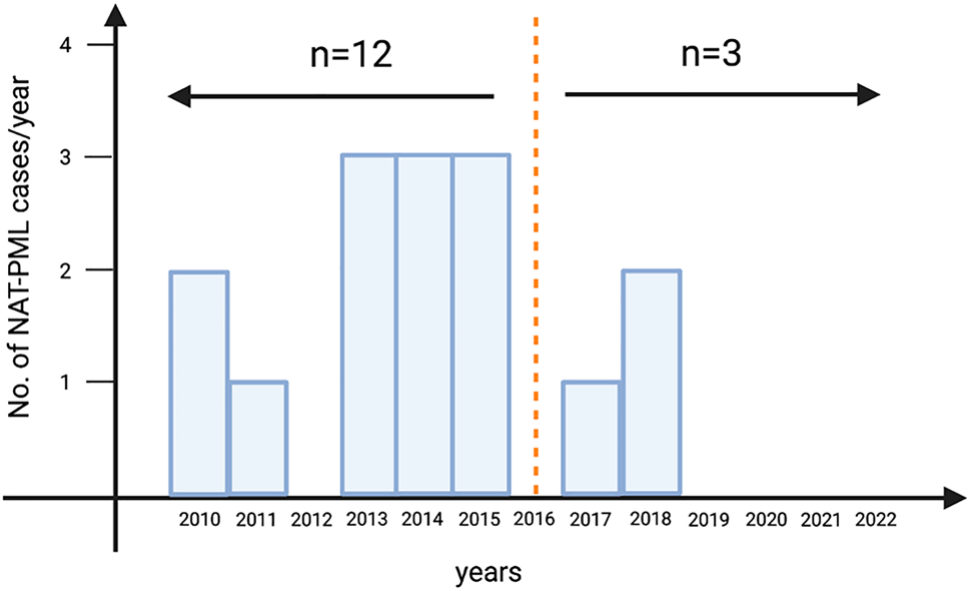
Time of occurrence of NAT-PML cases in Austria. Twelve cases were diagnosed before 2016, and only three thereafter. The vertical dashed line indicates the time of implementation of the risk stratification consensus guideline. *No.* number of, *NAT-PML* natalizumab-associated progressive multifocal leukoencephalopathy

As of December 2022, we identified 15 cases of NAT-PML. Ten patients (67%) were women, and the median age at PML diagnosis was 40 years (IQR 36–47 years). The demographics of the cohort are summarized in Table 1. Each patient had received a median of two immunotherapies before treatment with NAT, and two individuals were directly switched to NAT from second-line agents (cyclophosphamide and fingolimod (FTY)). One individual was treated with an immunosuppressant (cyclophosphamide) prior to NAT initiation. Patients had received a median of 52 (IQR 42–67) NAT infusions at the time of PML diagnosis. All but one patient (93%) had received more than 24, and seven (47%) more than 60 infusions. Four patients had no data on JCV serostatus or index at the time of NAT-PML diagnosis. Three of them were diagnosed with NAT-PML before the JCV index became available in 2012. The remaining individual was diagnosed with NAT-PML in 2013, but this patient’s JCV serostatus was not assessed. Among the 11 patients with available JCV antibody index, all but one had an index ≥ 2.8 at the last assessment before PML diagnosis (median JCV antibody index 3.5, IQR 3.0–3.6, range 0.4–3.7). The exception was a 55-year-old patient with a borderline pre-PML anti-JCV antibody index (0.4), who was diagnosed with PML after 20 NAT infusions. This patient’s JCV antibody index was tested again after PML recovery, two years after the latest pre-PML JCV assessment, and had increased to 2.8 (Supplementary Table 1). The number of new NAT-PML diagnoses decreased after 2015 compared to previous years. Overall, 12 (80%) cases occurred between 2010 and 2015 and only three (20%) in the years between 2016 and 2022 (Fig. 1).

**Table 1:** Patient characteristics and clinical outcome of the Austrian NAT-PML cohort (*n* = 15)

	No. (%) of patients	Median (IQR)	Mean (± SD)
Sex, female	10 (67)		
Age at PML diagnosis, y	15 (100)	40 (36–47)	41 (8)
MS duration at PML, y	14 (93)	10 (6–13)	10 (5)
RRMS at PML diagnosis	15 (100)		
No. of prior MS therapies	14 (93)	2 (1–2)	2 (1)
No. NAT infusions	15 (100)	52 (42–67)	55 (22)
Prior immunsuppression	1 (7)		
Diagnostic delay, d	15 (100)	42 (14–75)	53 (54)
Follow-up since PML diagnosis, m	11 (73)	76 (63–100)	81 (28)
EDSS pre-PML	15 (100)	3.5 (2.5–4.8)	3.7 (1.5)
EDSS post-PML	15 (100)	6.5 (4.0–8.8)	6.0 (2.8)
EDSS long-term outcome^#^	15 (100)	6.5 (4.0–9.3)	6.4 (3.0)
mRS at the end of the observation period	13 (87)	3.0 (3.0–5.0)	3.8 (1.7)
Deaths	3 (20)		
Acute NAT-PML phase	2 (13)		
Long-term follow-up	1 (7)		

*NAT* natalizumab, *PML* progressive multifocal leukoencephalopathy, *No.* number of, *IQR* interquartile range, *SD* standard deviation, *MS* multiple sclerosis, *RRMS* relapsing remitting multiple sclerosis, *y* years, *m* months, *d* days, *EDSS* expanded disability status scale, *mRS* modified Rankin scale

^#^Calculated from the last available EDSS of the entire cohort

### Features of the acute NAT-PML phase

At the time of PML diagnosis, all patients were in the symptomatic stage of the disease, and all had cerebral MRI characteristics compatible with PML. Moreover, JCV DNA was detected in the CSF of each patient during the diagnostic workup. Subsequently, all patients fulfilled the criteria for “definite” PML. The first MRI scan at PML suspicion showed widespread lesions in six (40%), unilobar affection in five (33%) and infratentorial involvement in six (40%) patients, respectively. PML lesions with Gd-enhancement at the time of PML suspicion were found in four patients and all four had a favorable short- and long-term outcome (median long-term EDSS increase of 0.5 (IQR − 0.3 to 1.1) compared to pre-PML; Table 2). Unilobar involvement on the first cerebral MRI was mostly observed among patients with severe EDSS increase (4/5). Quantitative measurement of CSF JCV load was available for 14/15 cases and ranged from 15 copies/µl to 3.1 Mio copies/µl. The median time from symptom onset to PML diagnosis was 42 days (IQR 14–75 days, range 2–180 days). All patients presented with at least two new neurological symptoms at the time of PML diagnosis. The most frequent symptoms at PML onset were gait and/or limb ataxia (73%) followed by cognitive and behavioral changes (67%). Seven patients (47%) presented with motor weakness and 27% had visual deficits. Five patients (33%) reported of a hitherto unknown type of headache. In one patient, severe headache was the only complaint for several weeks before developing a monoparesis, and the motor weakness aided in the diagnostic process. The cerebral MRI revealed a single Gd-enhancing lesion in the contralateral precentral area without oedema or mass effect. CSF examination showed a normal cell count, and JCV was detected by PCR for the first time in the CSF of the third lumbar puncture. In two patients (13%), epileptic seizures gave rise to the consideration of PML. Other deficits were speech or language disorders (40%), sensory abnormalities (20%), neglect, and incontinence (each 7%). Table 3 compares the survival rates and presenting symptoms to features from PML cohorts with different aetiologies [13, 16, 19–23]. Ataxia, sensory deficits and headache were most frequent in our study cohort.

**Table 2:** Patient characteristics, complications and administration of plasma exchange (PLEX) during PML among our patients stratified into mild (≤ 1.5; *n* = 6) and severe (> 1.5; *n* = 9) neurological worsening according to the EDSS^a^

#	MS duration, y	Age, y	No. NAT inf	pre-PML	EDSS	long-term	PML diagnostic delay, d	JCV index^b^	cMRI at PML suspicion				CSF	IRIS	Seizure	PLEX
					post-PML				Unilo-bar	Infratentorial	Widespread	Gd enhanc	JCV PCR^c^			
Mild disability progression according to EDSS change (≤ 1.5) at long-term																
1	13	33	68	1.0	1.0	0	47	3.6		+		+	22	+		
2	14	43	65	4.5	4.5	6.0	28	3.0	+			+	320	+		+
3	15	39	69	4.0	4.0	4.0	14	3.7		+		+	1700	+		+
4	N/A	48	94	3.5	4.0	4.0	7	3.5		+	+		25			+
5	9	41	27	2.5	3.5	3.5	60	N/A			+	+	+	+		+
6	19	48	96	3.5	4.5	4.5	42	3.5		+			15	+		
Severe disability progression according to EDSS change (> 1.5) at long-term																
7	6	38	48	6.0	6.5	8.0	14	3.4	+				7 × 10^5^		+	+
8	2	55	20	2.0	3.0	4.0	180	0.4	+				50	+	+	+
9	5	40	47	5.0	7.0	7.0^*^	2	N/A	+				94	+	+	+
10	10	37	41	6.0	8.5	9.0	150	N/A	+				3 × 10^6^	+	+	+
11	7	45	66	5.0	9.0	9.5	90	3.7			+		16,120	+		+
12	12	26	33	2.5	6.5	6.5*	45	N/A		+	+		300	+		+
13	10	35	61	2.5	10	10*	14	3.5		+			600	+		+
14	12	51	52	4.0	9.0	10	6	3.1			+		84,000	+	+	+
15	6	34	42	3.0	10	10*	90	2.8			+		50	+	+	+

*MS* multiple sclerosis, *y* years, *No* number of, *NAT* natalizumab, *inf*. infusions, *EDSS* expanded disability status scale, *PML* progressive multifocal leukoencephalopathy, *y* years, *d* days, *cMRI* cerebral magnetic resonance imaging, *Gd enhanc*. gadolinium enhancement, *CSF* cerebrospinal fluid, *PCR* polymerase chain reaction, *IRIS* immune reconstitution inflammatory syndrome, *PLEX* plasma exchange, *N/A* not available

^#^No long-term data available, therefore reflecting latest available EDSS

^a^EDSS change from pre-PML to long-term

^b^Latest available JCV index at the time of PML diagnosis

^C^In copies/µl

**Table 3:** Comparison of the presenting symptoms in HIV- and NAT-related PML patients and one pre-HIV cohort

	No	Survival*	Cognition/behavior	Paresis	Ataxia	Sensation	Speech/language	Vision	Headache	Seizure
NAT-PML (Moser 2023)	**15**	**87%**	**67%**	**47%**	**73%**	**20%**	**40%**	**27%**	**33%**	**13%**
PML pre-HIV (Brooks 1984)	109	< 20%	36%	33%	13%	–	27%	34%	7%	5%
PML HIV (Berger 1998)	144	9%	36%	42%	35%	19%	40%	19%	32%	9%
PML HIV (Berenguer 2003)	118	64%	N/A	70%	44%	19%	47%	20%	N/A	13%
NAT-PML (Clifford 2010)	28	71%^#^	43%	25%	14%	4%	18%	29%	–	18%
NAT-PML (Tan 2011)	40	> 70%^#^	54%	45%	–	7%	24%	41%	–	14%
NAT-PML (Mitsikostas 2014)	9	70%	67%	44%	22%	11%	33%	22%	11%	–
NAT-PML (Prosperini 2016)	39	92%	52%	36%	15%	–	3%	6%	–	6%
NAT-PML (Maillart 2017)	23	N/A	22%	44%	30%	N/A	17%	17%	N/A	N/A
NAT-PML (Blankenbach 2019)	142	91%	34%	34%	27%	11%	25%	16%	4%	3%

Text in bold marks the findings of the current study

*No*. number of patients included, *NAT-PML* natalizumab-associated progressive multifocal leukoencephalopathy, *PML* progressive multifocal leukoencephalopathy, *HIV* human immunodeficiency virus, *N/A* not available

*Survival of the acute PML phase

^#^Period of survival rate calculation not reported

All patients but two were treated with plasma exchange (PLEX) to expedite the removal of NAT and to restore immune surveillance within the CNS. Of the 14 patients (93%) receiving high-dose intravenous steroids, this treatment was started in 10 with the diagnosis of PML-IRIS. The remaining four had prophylactical steroid therapy during the acute PML phase and two of them did not develop IRIS. The additional therapeutic regimen was heterogeneous and included mirtazapine (*n* = 10, 67%), maraviroc (*n* = 7, 47%), mefloquine (*n* = 5, 33%) and cidofovir (*n* = 1, 7%).

### Short- and long-term outcome

The overall mortality rate was 20%. Two patients died of a fulminant PML-IRIS, both two months after the NAT-PML diagnosis, resulting in a survival rate of 87% at short-term follow-up. The pre-PML EDSS scores of these patients were 2.5 and 3.0, respectively. A third patient deceased at 56 years of age, five years after NAT-PML diagnosis at a nursing home. His pre- and post-PML EDSS scores were 4.0 and 9.0, respectively; and the cause of death is considered to be related to the fulminant neurological deterioration accumulated during NAT-PML.

**Fig. 2:**
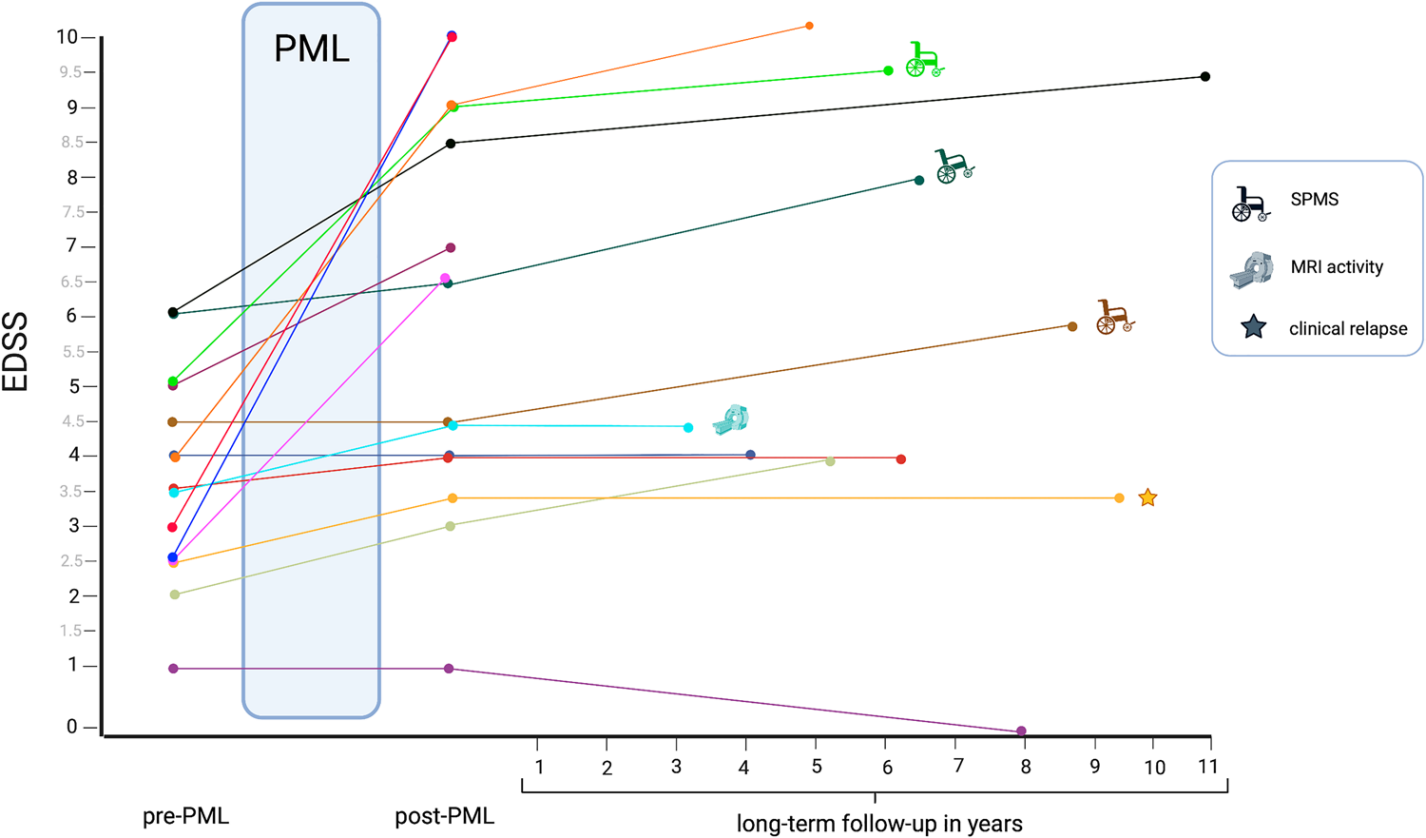
Short- and long-term outcome of each patient from the Austrian NAT-PML cohort. After PML recovery, we observed one clinical relapse and radiological disease activity in another patient, and three patients converted to SPMS. Overall, three patients died. *PML* progressive multifocal leukoencephalopathy, *SPMS* secondary progressive multiple sclerosis, *MRI* magnetic resonance imaging, *EDSS* expanded disability status scale

Two individuals were lost to follow-up shortly after the acute PML phase (their post-PML EDSS scores were 6.5 and 7.0, respectively). The median follow-up duration of the remaining 11 PML survivors was 76 months (IQR 63–100 months; range 41–132 months). The clinical course of each patient is shown in Fig. 2.

Regarding EDSS changes associated with the acute PML phase, seven patients (47%) had a poor outcome with severe disability progression at post-PML, and five (33%) worsened by ≥ 3 points on the EDSS compared to pre-PML. Six individuals (40%) developed epileptic seizures during the PML course, and 13/15 (87%) were diagnosed with PML-IRIS. Seizures were exclusively observed among patients with a severe EDSS increase on long-term outcome (Table 2).

Regarding the last available EDSS of the entire cohort (*n* = 15), severe accumulation of persistent neurological deficits compared to the pre-PML performance was observed in 60%. The overall median EDSS increased from 3.5 (IQR 2.5–4.8, range 1–6.0) at pre-PML to 6.5 (IQR 4.0–8.8, range 1–10) at post-PML and to 6.5 (IQR 4.0–9.3, range 0–10) at the end of the observation period. Among the six patients with only mild disability progression at long-term follow-up according to EDSS change, only one had a pre-PML EDSS score of > 4, compared to 4/9 patients among the cohort with poor outcome. The overall median mRS at the last follow-up was 3 (IQR 3–5, range 0–6). Excluding all deceased (*n* = 3), the median EDSS changed from 3.8 (IQR 2.5–5.0, range 1–6.0) at pre-PML to 4.5 (IQR 3.9–6.6, range 1–9.0) at post-PML and to 5.3 (IQR 4.0–7.3, range 0–9.5) at long-term. Excluding the deaths during the acute PML phase and the patients with lost to follow-up, the median EDSS of the remaining 11 patients remained stable during the extended observation period from post-PML (4.5; IQR 3.8–7.5, range 1.0–9.0) to long-term (4.5; IQR 4.0–8.5, range 0–10). However, the EDSS scores among those 11 PML survivors increased in six (55%) patients during long-term follow-up, remained stable in four (36%) patients and improved in one individual.

After PML recovery, 7/11 (64%) patients resumed immunotherapies. The initial approaches consisted of intravenous immunoglobulins (IVIG) every 4 weeks in three patients, while two received glatiramer-acetate (GA), one the anti-CD20 monoclonal antibody rituximab and another one dimethyl fumarate (DMF). Apart from the case in which anti-CD20 treatment was initiated 3.5 years after PML, all immunotherapies were started within 12 months from the PML diagnosis. Throughout the long-term observation, one patient continued receiving GA, DMF and IVIG, respectively, while the initial treatment was stopped in two other patients (on IVIG and anti-CD20 therapy) without resuming additional immunotherapies. Of the two individuals switched to other immunotherapies during follow-up, one patient receiving GA had a clinical relapse at 12 months from PML and was then escalated to FTY. At the last available follow-up seven years later, this patient continued FTY therapy and had remained free of inflammatory MS-related disease activity and without EDSS progression. The second patient was treated with monthly IVIG for twelve months after PML recovery and was then subsequently switched to receive pulsed steroid-therapy for three additional months due to an EDSS increase of 0.5 points. Thereafter, this patient had no further immunotherapy and the EDSS remained stable at the last follow-up 9 years later. One patient receiving IVIG infusions for 24 months remained without immunotherapy thereafter. This patient converted to SPMS shortly after PML recovery with a pre-PML EDSS of 6.0 and a long-term EDSS of 8.0. The anti-CD20 therapy was initiated in a patient with no therapy due to continuous clinical deterioration in the years following PML recovery and transition to a secondary progressive course (SPMS). This patient received a total of five anti-CD20 infusions at six-month intervals and the EDSS increased from 4.5 at post-PML to 6.0 at long-term follow-up. Overall, inflammatory MS disease activity following PML recovery was rare. Besides one single clinical relapse recorded, signs of inflammatory MRI activity were observed in one other individual. This patient had no immunomodulatory treatment and the cerebral MRI performed at two years from PML showed Gd-enhancement of two intracranial MS lesions. No immunotherapy was started, and the individual remained clinically stable with an EDSS of 4.5. Three patients entered a progressive MS course during the follow-up, of whom two subsequent to PML recovery and the remaining at three years from PML. SPMS was diagnosed after a median of 7 years (IQR 7–12 years) from the initial MS diagnosis and at a median age of 45 years (IQR 42–46 years). Of note, while the EDSS of majority of the PML survivors increased, the EDSS of the patient treated with DMF improved during the extended observational period. This patient had only clinical neurological signs of MS without disability at pre-PML and at post-PML (EDSS 1.0) and fully recovered at long-term follow-up (EDSS 0). During PML, this patient had impaired cognition, vision, and coordination.

The JCV antibody index values were repeated in three patients during long-term follow-up and are shown in the Supplementary Table 1.

## Discussion

No therapeutic recommendations exist for the management of the post-NAT-PML period, and the impact of cerebral JCV infection on the MS course is unclear. In this nationwide study, we analyzed the long-term outcome of NAT-PML during a follow-up of up to 132 months and a median of 76 months. We found that many patients had accumulated severe persistent neurological deficits at long-term follow-up, which was however mostly related to the EDSS changes associated with the acute PML phase. The median EDSS substantially increased from 3.5 at pre-PML to 6.5 at post-PML and 6.5 at long-term. In our cohort, we found that patients with a pre-PML EDSS > 4 were more likely to accumulate severe EDSS progression at long-term follow-up, which might indicate that individuals with lower disability at the time of NAT-PML diagnosis have a better prognosis. At the end of the observation period, only one individual had an EDSS score below 3.5. This patients’ EDSS even decreased from 1.0 at pre- and post-PML to 0 at long-term follow-up. Importantly, one third of this cohort had substantial neurological worsening associated with the acute PML phase with an EDSS increase of ≥ 4 score points. Our data therefore emphasize that cerebral JCV infection represents a devastating disorder that must be avoided at all costs. Among the 12 PML survivors, the median EDSS increased by 0.8 points during the observational period after PML recovery. Importantly, 55% of the patients with available follow-up worsened in the EDSS at long-term assessment compared to post-PML. The median EDSS change of 0.8 has to be interpreted in the context of the nonlinear relationship of the score differences and the clinical performance in the upper EDSS part. Of note, the baseline characteristics of our cohort at the time of PML diagnosis suggest that some patients were in the progressive MS stage already as 7/15 had an EDSS between 4.0 and 6.0. At that level, any increase in the EDSS results in disproportionate disability accumulation. Nevertheless, the low rate of inflammatory MS disease activity in our cohort during long-term follow-up is unexpected. We recorded a single relapse in the entire study population following PML recovery and MRI activity was present in one other individual. Interestingly, attenuation of inflammatory processes after PML recovery does not seem to be related to the use of immunotherapies. In fact, only two patients were treated with high efficacy DMTs after PML, while the majority from our cohort remained either without immunomodulatory substances, or received first-line agents, IVIG or steroids. Of note, IVIG were used historically in Austria as off-label treatment in MS and cannot be recommended. Other studies confirm that the EDSS plateaus at six months post-PML [24] and remains stable throughout the further follow-up of 24 months [24, 25]. This is especially remarkable as NAT is mostly used for the treatment of patients with highly active MS after insufficient response to first-line DMTs [26, 27]. Moreover, NAT discontinuation is frequently associated with the rebound phenomenon, which is characterized by the return of disease activity to pre-NAT levels [28]. Admittedly, the median pre-PML EDSS score of 3.5 of our study cohort may suggest that, by the time PML occurred, neurodegenerative more than inflammatory processes may already have been prominent in some individuals [29]. Whether degenerative processes in turn are accelerated by cerebral JCV infection remains to be further elucidated. Indeed, among the PML survivors of our cohort, 25% were diagnosed with SPMS within three years from PML and after a median of 7 years from the initial MS diagnosis. Lastly, three patients died during or after NAT-PML, yielding a long-term survival rate of 80% among our study cohort.

The timing of re-initiation and the selection of any DMT after NAT-PML raises safety and/or efficacy concerns. A retrospective study comprising 23 NAT-PML survivors analyzed the impact of injectables and that of DMF (*n* = 5) and of FTY (*n* = 9) on the (post-) PML MS course [25]. DMTs were started on average seven months after NAT withdrawal and relapses were only observed among patients on injectables. Moreover, no patient had signs suggestive of a clinical or radiological reactivation of PML throughout the two-year follow-up. In line, we did not observe clinical or radiological signs suggestive of a spontaneous deterioration after recovery from NAT-PML. To conclude, the available evidence on the MS treatment approaches in PML survivors, comprising two medium-sized retrospective NAT-PML cohorts including our own [25], indicates that once the immune surveillance is restored, a reactivation of PML is unlikely despite re-initiation of immunotherapies. While no controlled trails exist on the MS treatment approaches following PML, the available data have found no additional safety concerns that might affect the selection of the DMTs after NAT-PML recovery.

Since no specific treatment for PML exists, preventive strategies play a paramount role in the management of patients with long-term NAT exposure. In fact, adherence to the risk mitigation plan, early switching of patients at high-risk to other DMTs as well as implementation of extended interval dosing (EID) have contributed to reduce incidence rates of NAT-PML over the last years [30–34]. In line, we show that the frequency of NAT-PML clearly declined and that only 20% of the cases occurred after implementation of the risk stratification guideline in 2016 [11]. Since then, a dropping number of NAT prescriptions and a high rate of treatment discontinuation among the JCV seropositive population have been reported in Austria [27, 35]. According to the established stratification algorithms, only one Austrian NAT-PML case would have been classified to be at low risk. However, this patient was > 50 years old, supporting that immunosenescence constitutes an additional independent risk factor for opportunistic infections [36, 37] and, more specifically, for NAT-PML [13, 37]. A narrowed T-cell repertoire and impaired T-cell functions in the aging immune system explain the rationale behind the immunosenescence theory, resulting in inappropriate cytotoxic T-cell activation that ultimately hampers viral clearance [38–40]. Therefore, once patients advance in treatment duration and age, the beneficial effect on disease control and the risk of rebound after stopping NAT [41] have to be weighed against increasing PML risk [42].

A reliable marker for early diagnosis of NAT-PML would be highly appreciated, considering the significant diagnostic delays observed in the Austrian cohort. PML was diagnosed after a median of 53 days after symptom onset in our cohort, similar to the reported time frames of 69 and 49 days from other studies comprising 40 and 39 NAT-PML patients, respectively [14, 16]. Together, these findings underline that some patients continue receiving NAT despite the presence of PML-related symptoms. Notably, relapses during NAT treatment are rare [43], and new clinical signs should prompt clinicians to rule out PML. However, PML can present with a variety of neurological features depending on the anatomical localization of the lesions, and deficits can be discreet at onset [44]. Compared to the presenting symptoms from most other NAT-PML cohorts [13, 16, 21, 22], we found a higher frequency of cognitive disturbances (67%) and importantly, 33% of our patients complained about a new type of headache during PML. Headache is highly prevalent among MS patients, who primarily suffer from migrainous and medication overuse subtypes [45]. Intriguingly, headache is a common feature of HIV-PML [20], but has not frequently been associated with NAT-PML [13, 16, 21–23]. One patient in our cohort who presented with headache as a core complaint had no explanatory MRI lesion. Therefore, patients and physicians should be alerted that headache can be the first symptom of PML in NAT-treated individuals. Epileptic seizures, which are clinically more emergent signs, occurred in 40% of our study population and were consistently associated with a severe EDSS increase on long-term outcome.

While clinical vigilance is indispensable for early diagnosis of symptomatic PML, paraclinical examinations may identify PML during its asymptomatic stage. The *Italian PML group* revealed that PML suspicious lesions were retrospectively detectable in 78% of the pre-diagnostic MRI scans [46]. PML compatible abnormalities appeared on average five months (yet up to 11 months) before the clinical onset [46]. This is important, as early PML diagnosis during the asymptomatic phase not only prevents further NAT infusions but also impacts the outcomes [14, 21]. In a cohort of 588 symptomatic and 90 asymptomatic NAT-PML patients, the survival rate was 73% and 92%, respectively [47]. Surprisingly, none of the Austrian NAT-PML cases were diagnosed before the onset of clinical symptoms. The acute PML survival rate of our study (87%) is consistent with that of other NAT-PML cohorts (70–92%) and higher compared to historical PML cohorts and to HIV-PML prior to the introduction of the highly active antiretroviral therapy (HAART) (Table 3) [16, 20–23, 48–51]. Restoration of immune competence by HAART however not only substantially decreased the prevalence of opportunistic infections in HIV patients, but also resulted in an improved survival of HIV-PML [51–53]. In fact, in a large cohort of 118 HIV-PML patients, about two-thirds survived the median observation period of 2.2 years, approximating survival rates of NAT-PML [54]. Lastly, we corroborate findings from an Italian cohort that patients with cerebral Gd-enhancement at an early stage may experience a milder PML course [21]. Gd-enhancement is consistent with an inflammatory PML course, but it also represents a core feature of IRIS. PML-IRIS occurs in approximately 90% of all PML patients, mostly at later stages of the disease, frequently after NAT removal or PLEX [13, 14, 55, 56]. The distinction between inflammatory PML and PML-IRIS appears to be relevant for the treatment strategy. While the mainstay for acute NAT-PML treatment consists of PLEX to accelerate NAT clearance and restore the migratory function of lymphocytes, steroids might mitigate the fulminant inflammatory response to the infectious pathogen leading to IRIS [57]. Importantly, new approaches for the treatment of acute cerebral JCV infection are being investigated that will hopefully improve the outcomes of NAT-PML.

Main limitations of this study stem from its retrospective design and the ordinal rating system EDSS, which we used as main outcome score. Also, two patients were lost for follow-up shortly after recovery from PML. Furthermore, we could not perform comparative statistical analyses of subgroups due to the small number of cases available.

## Conclusion

Along with risk mitigation strategies and generally lowering rates of patients treated with NAT, the number of NAT-PML cases is decreasing over time. Among this cohort, NAT-PML was associated with severe persistent neurological deficits in the majority of cases. During the follow-up of up to 132 months after NAT-PML, inflammatory MS disease activity was a rarity, but a major proportion of patients had a progressive EDSS increase indicative of ongoing neurodegenerative disease processes. Whether our findings may suggest a modification of MS course beyond natural course needs to be investigated in larger cohorts enabling adjustment for confounders.

### Supplementary Information

Below is the link to the electronic supplementary material.

Supplementary file1 (DOCX 15 KB)

## Data Availability

The data that support the findings of this study are available on request from the corresponding author (J.S.).
