# Supplementary material for: Long-term outcome of natalizumab-associated progressive multifocal leukoencephalopathy in Austria: a nationwide retrospective study
Source: J Neurol. 2023 Sep 20;271(1):374–85. doi: 10.1007/s00415-023-11924-7 (PMC10770206; doi:10.1007/s00415-023-11924-7)
Supplement: Supplementary file 1 — Supplementary file1 (DOCX 15 KB) [file 415_2023_11924_MOESM1_ESM.docx]

Journal of Neurology

**Long-term outcome of natalizumab-associated progressive multifocal leukoencephalopathy: a nationwide retrospective study**

Tobias Moser, Georg Zimmermann, Anna Baumgartner, Thomas Berger, Gabriel Bsteh, Franziska Di Pauli, Christian Enzinger, Elisabeth Fertl, Thomas Heller, Stefan Koppi, Paulus S. Rommer, Georg Safoschnik, Thomas Seifert-Held, Robert Stepansky, Johann Sellner*

* Correspondence: Prof. Dr. Johann Sellner MBA FAAN FEAN, Department of Neurology, Landesklinikum Mistelbach-Gänserndorf, Liechtensteinstrasse 67, 2130 Mistelbach, Austria. Tel. +43-2572-9004-12850. FAX +43-2572-9004-49281. E-mail address: johann.sellner@mistelbach.lknoe.at.

**Supplementary Table 1** JCV antibody (ab) index course of three patients (#2,4,8) before and after NAT-PML

| # | JCV ab index  at pre-PML | JCV ab index  after PML recovery | Time between PML diagnosis and follow-up JCV ab index assessment in years |
| --- | --- | --- | --- |
| 2 | 3.0 | 4.0 | 5 |
| 4 | 3.5 | 2.3 | 1 |
| 8 | 0.4 | 2.8 | 2 |

NAT-PML= natalizumab-associated progressive multifocal leukoencephalopathy; PML = progressive multifocal leukoencephalopathy
